# Childhood exposure to physical and emotional violence over a 57-year period in Sweden

**DOI:** 10.1177/14034948211023634

**Published:** 2021-06-24

**Authors:** Steven Lucas, Staffan Janson

**Affiliations:** Department of Women’s and Children’s Health, Uppsala University, Sweden

**Keywords:** Child physical abuse, child emotional abuse, child maltreatment, corporal punishment, primary prevention, epidemiology, survey studies, prevalence studies, gender differences

## Abstract

**Aims:**

The aim of the present study was to examine the prevalence of childhood experiences of physical violence (CPV) and emotional violence (CEV) at the hands of parents over a 57-year period among adults born between 1937 and 1993.

**Methods:**

In 2012, a survey among women and men aged 18–74 years in Sweden was undertaken to examine the lifetime prevalence of physical, psychological and sexual violence and associations with current health in adulthood. Questionnaires were based on the Adverse Childhood Experiences study and a previous national survey of violence exposure. Descriptive statistics were used to analyse the frequency of exposure to CPV and CEV, and changes over time were analysed using analysis of variance and logistic regression.

**Results:**

A total of 10,337 individuals participated (response rates: 56% for women and 48% for men). CPV decreased significantly over the time period studied, particularly for those born after 1983. This decrease was more evident for male respondents. Throughout the time period studied, the proportion of women reporting CEV was higher than for men. Among both genders there was a steady rise in CEV rates from those born in the late 1930s to those born in the mid-1980s, after which there was a decline that was more marked for men.

**Conclusions:**

A significant group of children in Sweden experience violence at the hands of parents. However, our data corroborate previous national studies that children’s exposure to violence has decreased. Clear gender differences indicate that these changes have affected girls and boys differently.

## Introduction

Globally, physical and emotional abuse affect a substantial proportion of the world’s children, with far-reaching consequences for the health and development of those who are exposed [1–3]. There is a growing body of evidence that the detrimental effects of exposure to violence during childhood underlie much of the burden of physical and mental health problems throughout the lifespan [3,4]. The grave impact of child maltreatment has given rise to a number of global initiatives to understand and eradicate its root causes – efforts that involve both the identification of risk factors and preventive efforts to alleviate them [5,6].

The use of physical and emotional violence against children represents a spectrum that includes severe forms of abuse as well as painful or humiliating acts that are accepted as forms of discipline in many societies [7]. Correlations have been found between the use of corporal punishment (CP) against children and other forms of physical violence experienced during childhood [8], and a number of studies have indicated that physical punishment in itself has similar detrimental effects [9,10]. This has led to an increase in the number of countries worldwide that have adopted legislation against CP. It is also becoming increasingly evident that CP is ineffective in creating positive changes in child behaviour [11]. In 1979, Sweden was the first country to adopt a ban on CP, and many more nations have since enacted similar laws.

Repeated studies among 15-year-old youth in Sweden have shown a decrease in lifetime experiences of physical violence at the hands of a parent from 35% in 1995 to 12% in 2016 [12]. A similar decline in physical abuse was recently reported among 15- to 17-year-old students in serial school-based surveys between 2008 and 2017 [13]. These decreases have not been reflected in official statistics from law enforcement or social services reports of physical abuse, which instead have shown stable or increasing frequencies over the past decades [14].

In 2012, a survey study among women and men aged 18–74 years in Sweden was undertaken to examine the lifetime prevalence of physical, psychological and sexual violence and associations with current health in adulthood [15]. This data set provides the opportunity to study the prevalence of adults’ reported experiences of violence at the hands of parents and other adults during childhood, including acts of CP, over a 57-year period (birth year 1937–1993).

### Aims

The aim of the present study was to analyse the prevalence of childhood experiences of physical and emotional violence at the hands of parents over a 57-year period with respect to changes over time as well as differences between women and men.

## Methods

The survey was carried out in the spring of 2012. As no existing survey instrument included all the areas we wished to examine, a questionnaire was designed based on the Adverse Childhood Experiences (ACE) study and a previous national violence prevalence study in Sweden [3,16]. The first section concerns present socio-demographic information, followed by items regarding family conditions during childhood, including physical and emotional neglect as well as parental substance abuse, mental illness, suicide attempts and criminal behaviour. Sexual, physical and psychological violence of varying severity were inquired about separately for exposure at ages 0–15, 15–17 and >18. For exposure before the age of 18, identical but separate sections addressed violence perpetrated by adults as opposed to peers for each type of violence. Other question sections included behaviours related to present health as well as current physical and mental health.

Face validity and content validity of the questionnaire and all questions included were evaluated at Statistics Sweden (SCB) with the use of expert review, as well as cognitive interviews with people with and without a history of violence exposure. Minor adjustments were then made, and the questionnaire was piloted among 2000 individuals, 1000 of whom received a version with half as many questions to evaluate whether the length of the instrument would affect the response rate. No differences were seen between the groups with respect to response rate or item non-response. The final Violence and Health Survey instrument consisted of 97 questions in the Swedish language, with more than 300 sub-items [17].

Questionnaires were distributed to 10,000 women and 10,000 men randomly selected throughout Sweden. Only individuals with a Swedish personal identification number residing in Sweden were included in the population sample. As the questionnaire was only available in Swedish, participation required sufficient knowledge of the language to understand the questions and response alternatives. First, an introduction letter was sent, informing about the study and its focus, that the individual could opt not to participate by contacting the research group or SCB and that they otherwise would be contacted again within a week. The subsequent letter gave instructions about completing the survey online, and paper questionnaires were sent to those who did not respond online. Four reminders were sent.

Respondents were informed that by completing the online or paper version of the questionnaire, they gave their informed consent for their data to be handled as described above. In total, 10,337 individuals participated in the survey, giving a response rate of 52% (56% for women, 48% for men), with low levels of item non-response. An analysis of non-responders was performed, taking socio-demographic factors and welfare-based variables into account, and a multifactorial weighting algorithm was created to correct for under-represented respondent groups [18].

The study was approved by the Ethical Review Board in Uppsala (Dnr 2011/156).

### Outcome variables

As in many other previous studies, child physical abuse (CPA) and emotional abuse (CEA) were operationalised through questions reflecting specific acts of violence. The outcome variables used were based on the following questions posed separately for those aged 0–14 and those aged 15–17: ‘Sometimes girls/boys experience psychological or physical violence from an adult. It could be from parents, relatives, neighbours, school/preschool personnel, or other known adults or strangers. About how often did it happen before you turned 15 (or “when you were 15–17”) that an adult did any of the following to you? (a) humiliated or oppressed you verbally (put you down, insulted or degraded you), (b) threated to hurt you, (c) slapped you, pulled your hair, shoved you or shook you so it hurt, (d) punched you, hit you with an object, kicked you or choked you, (e) harmed you with a knife or gun, (f) exposed you to any other type of violence?’. The response options were ‘never’, ‘once’, ‘sometimes’ or ‘often’. The variable for childhood physical violence (CPV) was positive for any response other than ‘never’ to items (c), (d), (e) or (f). The variable for childhood emotional violence (CEV) was positive for any response other than ‘never’ to item (a). A follow-up question regarding the perpetrator of the CPV or CEV was worded as ‘Which adult(s) did this to you?’, with the response options ‘father/stepfather/mother’s partner’, ‘mother/stepmother/father’s partner’, ‘male adult relative’, ‘female adult relative’, ‘other adult male you know’, ‘other adult female you know’, ‘male stranger’ or ‘female stranger’. Responses including ‘father/stepfather/mother’s partner’ or ‘mother/stepmother/father’s partner’ were used to create the outcome variables CPV from a parent and CEV from a parent.

### Data analysis

Data were analysed using IBM SPSS Statistics for Windows v27 (IBM Corp., Armonk, NY). Weighted data were applied for descriptive statistics in order to adjust for under-represented groups in the responding population. Frequencies of the outcome variables were calculated for each birth year and transformed into time series, which were then subjected to T4253H smoothing for graphical presentation.

One-way analysis of variance (ANOVA) was performed using the time series data to study associations between the respondents’ birth year and the proportion of respondents exposed to CPV or CEV in each respective birth year. Visually apparent increases or decreases in outcome variable frequencies were further analysed using logistic regression, with exposure to CPV or CEV as the dependent variable and birth year as the independent variable. For this purpose, a dummy variable was created by dividing the respondents into six 10-year groups according to birth year. The logistic regressions were performed separately for women and men using non-weighted data. A significance level of <0.05 and two-tailed analyses were applied throughout.

## Results

**Figure 1.**
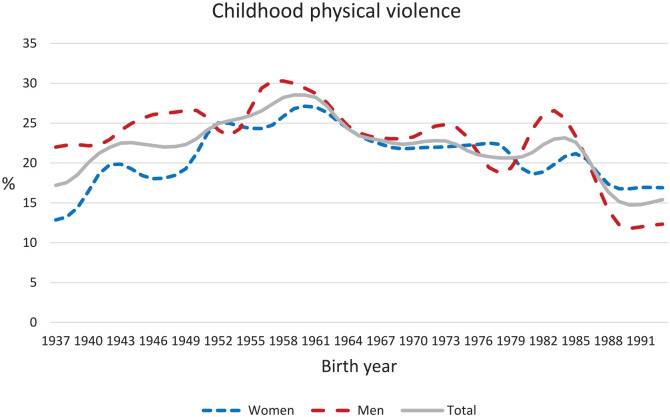
Time series depicting percentage of respondents born between 1937 and 1993 reporting childhood exposure to any kind of physical violence by a parent.

**Figure 2.**
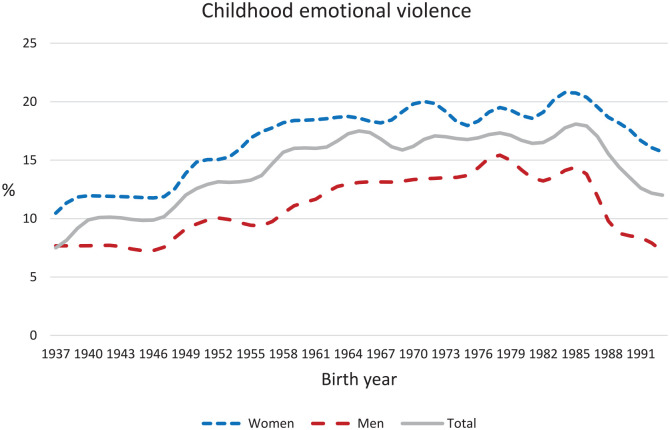
Time series depicting percentage of respondents born between 1937 and 1993 reporting childhood exposure to any kind of emotional violence by a parent.

In total, 21.7% of women reported exposure to CPV and 18.0% CEV from a parent before the age of 18. Women reported that CPV was perpetrated by fathers (15.6%) and mothers (12.9%) to a similar extent, which was also true for CEV (11.8% by fathers and 10.4% by mothers). Exposure to CPV was reported by 23.3% and exposure to CEV by 11.5% of the men. Among men, fathers were the perpetrator nearly twice as often as mothers for both CPV (19.9% vs. 9.9%, respectively) and CEV (9.2% vs. 4.9%, respectively). The frequencies of CPV and CEV over the period 1937–1993 are shown in Figures 1 and 2.

There was an apparent increase in the proportion of women reporting CPV from birth year 1937 to 1953, a peak around birth year 1960 and a slow decline thereafter. For men, these tendencies were less pronounced, although a steep decline in CPV prevalence was seen for those born after 1984.

Throughout the entire time range, women reported exposure to CEV to a greater extent than men did. For both women and men, there was a gradual net twofold increase in CEV prevalence from birth year 1937 to 1985, with a decline thereafter that was more marked for men.

ANOVA showed a significant association between birth year and CPV exposure among men (*F*(1, 55)=10.44, *p*=0.002) but not among women (*F*(1, 55)=0.003, *p*=0.956). CEV was significantly associated to birth year among women (*F*(1, 55)=30.198, *p*<0.001) and among men (*F*(1, 55)=7.907, *p*=0.007).

As shown in Table I, logistic regression indicated that birth year was a significant predictor of CPV among both women and men, with lowest odds of exposure for the respondents born between 1984 and 1993.

**Table I.** ORs (95% CIs) from logistic regression analyses for exposure to physical violence by a parent using respondents’ birth year in 10-year groups as the independent variable.

	Women			Men		
	OR	95% CI	*p-value*	OR	95% CI	*p-value*
*Block 1: birth year in decade groups*						
Birth year 1984–1993 (reference)			0.002			<0.001
Birth year 1974–1983	1.1	0.8–1.4	0.585	1.6	1.2–2.2	0.002
Birth year 1964–1973	1.3	1.0–1.6	0.046	1.7	1.3–2.3	<0.001
Birth year 1954–1963	1.5	1.2–1.8	0.001	2.3	1.7–3.0	<0.001
Birth year 1944–1953	1.2	0.9–1.5	0.201	1.9	1.5–2.5	<0.001
Birth year 1937–1943	0.9	0.7–1.2	0.601	1.5	1.1–2.0	0.023

OR: odds ratio; CI: confidence interval.

Logistic regression for CEV (Table II) showed a somewhat different picture. Although birth year overall was a significant predictor, women born between 1944 and 1953 and between 1937 and 1943 had lower odds of CEV than those born between 1984 and 1993, and men born between 1974 and 1983 had significantly higher odds of CEV compared to those born between 1984 and 1993, while those born between 1937 and 1944 had significantly lower odds.

**Table II.** ORs (95% CIs) from logistic regression analyses for exposure to emotional violence by a parent using respondents’ birth year in 10-year groups as the independent variable.

	Women			Men		
	OR	95% CI	*p-value*	OR	95% CI	*p-value*
*Block 1: birth year in decade groups*						
Birth year 1984–1993 (reference)			<0.001			<0.001
Birth year 1974–1983	1.0	0.8–1.2	0.680	1.5	1.1–2.0	0.023
Birth year 1964–1973	1.0	0.8–1.2	0.857	1.3	1.0–1.9	0.074
Birth year 1954–1963	0.9	0.7–1.1	0.305	1.0	0.7–1.4	0.938
Birth year 1944–1953	0.7	0.5–0.9	0.001	0.9	0.6–1.2	0.476
Birth year 1937–1943	0.5	0.4–0.7	<0.001	0.6	0.4–1.0	0.034

## Discussion

Our findings indicate a significant decrease in parent-perpetrated CPV against children <18 years of age in Sweden for those born after 1983. This is well in line with serial studies in 1995, 2006, 2011 and 2016 among youth in Sweden, which showed a steady decline in rates of CPV from 35% to 12% [12]. In contrast, a study comparing two surveys from 2003 and 2008 in the USA showed a decrease in youth reporting CEV but not CPV by a caregiver [19]. Our findings regarding CEV are less clear. Although a decline was evident for men born after 1983, there was a striking increase in the prevalence of CEV from those born in 1937 to those born in 1986. We have not seen this reported in the literature previously. One contributing factor may be that the concept of CEV as a distinct form of child abuse is considerably newer compared to CPV, which may also be reflected in individuals’ readiness to identify emotionally abusive actions as such [20].

Although reported rates of child sexual abuse often are stratified by gender, fewer studies have reported prevalence rates for CPV and CEV separately among women and men [21]. The difference we found between women and men regarding the overall prevalence of CEV is in line with one survey study from the USA, indicating that women experienced significantly higher rates compared to men [22]. Another study showed only slightly higher rates of CEV among women (35.7%) compared to men (32.2%) [23].

The decrease in exposure to CPV and CEV among the respondents over the time period studied may have many explanations, including changes in societal factors such as increased gender equality between mothers and fathers in the workplace and at home, increased universal access to high-quality childcare and preschool and a gradual transition in parental and societal perceptions of children towards being competent individuals and bearers of rights and autonomy [14,24]. The decrease in CPV and CEV in our results also coincides with the decades following the Swedish ban on CP. Whether the ban had a direct effect on the prevalence of CP or child abuse is not clear. The legislation came at a time when parents’ attitudes already had shifted towards less acceptance. In the 1960s, >65% of Swedish parents approved of CP, and many considered it to be an important part of childrearing. This proportion had decreased to <50% by 1980. Research in Sweden has confirmed a continued decrease in parents’ self-reported use of CP, especially between 1980 and 2000, which is in line with our findings among individuals born during that period [24]. A 2011 survey showed that only 7% of parents were in favour of using CP, and 5% responded that they had actually used it during the previous year [25].

A similar but later change in parental attitudes and behaviour towards children has occurred in Finland, the second country to outlaw CP of children in 1984. The later decrease in Finland may have been due to some uncertainties about the law that were made clearer in a 1992 Supreme Court statement [26]. It should also be kept in mind that substantiated child abuse has declined during the last decades in countries without a ban on CP, including the USA. Such trends have been attributed to a number of social changes such as declines in divorces, births among unmarried women and households headed by a single mother [27,28].

The decreases seen in CPV are in stark contrast to data on police reports of child abuse among children aged 0–6 years perpetrated by a person known to the child, which increased fivefold between 1982 and 2000 and has continued to rise sharply [29,30]. According to the Swedish National Board for Crime Prevention, these increases do not represent an increase in child abuse per se, but rather increased public awareness of child abuse as a criminal offence as well as a greater propensity for preschools and schools to report suspected abuse to the social services, which in turn have been more inclined to report such suspicions to the police [31]. This points out the presence of a large proportion of child maltreatment that is undetected and underscores one of the important limitations of using informant data from, for example, law enforcement, social services or health care when estimating the prevalence of child maltreatment, as these data represent only the tip of the iceberg [21,32].

### Strengths and limitations

The strengths of the present study include the use of a large population-based sample, a thorough pilot study prior to data collection and extensive non-response analysis. Although the survey instrument used was a new construct, the questions included were based closely on those used in extensively applied instruments including the ACE questionnaire, and cognitive interviews indicated good face and content validity. Despite the aim of gathering detailed information about the experiences of the respondents, the questions posed provide limited response options to complex issues and may thus have narrowed the information that was obtained. Due to the cross-sectional design of the study, the conclusions that can be drawn are limited to associations between the observed phenomena, and assumptions cannot be made about cause-and-effect relationships. As the survey was carried out in 2012, recent developments may have occurred in the prevalence of violence exposure, which should prompt a follow-up of this survey. Different types of potential bias, including recall bias and social acceptability bias, must be acknowledged, the extent and effects of which cannot be estimated. Postal surveys do not reach the most marginalised people in society, including the homeless and incarcerated. We may expect that the levels of violence might be higher if individuals from these groups were included.

## Conclusions

Sweden has been a benchmark country in realising child rights and decreasing children’s exposure to violence [2]. Our data corroborate previous national studies among youth and parents over the last four decades that support this progress. While the great majority of parents in Sweden repudiates the use of violent or humiliating upbringing practices, a significant group of children still experience CPV and CEV at the hands of their parents. It is therefore important not only to maintain but also to intensify preventive efforts aimed at identifying and addressing children at risk of maltreatment early before violence has occurred.
